# Increased uric acid to high-density lipoprotein ratio positively correlated with stroke risk

**DOI:** 10.3389/fneur.2025.1577077

**Published:** 2025-06-02

**Authors:** Tieshi Zhu, Yong He, Erxinxian Bei

**Affiliations:** ^1^Department of Neurology, Zhanjiang Central Hospital, Guangdong Medical University, Zhanjiang, China; ^2^Department of Neurology, Liuyang Jili Hospital, Changsha, China; ^3^Department of Neurology, Haikou Affiliated Hospital of Central South University Xiangya School of Medicine, Haikou, China

**Keywords:** UHR, stroke, NHANES, cross sectional study, public health

## Abstract

**Background:**

The Uric Acid-to-HDL Ratio (UHR), a novel index derived from serum uric acid and high-density lipoprotein, has been linked to hypertension and poor diabetes control. It has also been shown to predict ischemic heart disease and is strongly associated with collateral circulation and coronary artery flow reserve. However, fewer studies have focused on the relationship between UHR and stroke, highlighting the need for further research in this area.

**Methods:**

The study included 33,192 individuals from the NHANES 1999–2023, of whom 1,363 had a history of stroke. The nonlinear relationship between UHR and stroke risk was assessed using restricted cubic spline (RCS) analysis, and the robustness of the findings was further tested through stratified analysis. Logistic regression was employed to analyze the relationship between UHR and stroke risk, considering both UHR as a continuous variable and its categorization into quartiles (Q1–Q4).

**Results:**

UHR was not nonlinearly associated with stroke (*p* for overall <0.01; *p* for nonlinearity = 0.65), and the RCS graph approximated a straight line with a positive slope. UHR was significantly associated with an increased risk of stroke, both when analyzed as a continuous variable (Model 4: OR = 1.02, 95% CI 1.01–1.03, *p* < 0.01) and when categorized into quartiles (Q4, OR = 1.31, 95% CI 1.11–1.55, *p* < 0.01).

**Conclusion:**

There was a significant positive correlation between UHR and stroke risk.

## Introduction

Stroke remains a leading cause of mortality and disability worldwide, placing a substantial burden on individuals, families, and healthcare systems (1, 2). Given its unfavorable prognosis and the long-term functional impairments experienced by many survivors, a comprehensive understanding and identification of risk factors are essential for effective prevention strategies (3–5).

Traditional risk factors for stroke include hypertension, diabetes mellitus, dyslipidemia, smoking, and alcohol consumption (6–10). Beyond these established factors, elevated serum uric acid (UA) and reduced high-density lipoprotein (HDL) levels have also been implicated in stroke pathogenesis (11, 12). The Uric Acid-to-HDL Ratio (UHR) is a novel and easily accessible index that integrates these two parameters, providing a potential marker for systemic inflammatory and metabolic conditions (13, 14). Studies have demonstrated that UHR outperforms serum uric acid and HDL alone in predicting coronary artery disease and the severity of coronary stenosis (15). Furthermore, UHR has been associated with an increased risk of type 2 diabetes mellitus, metabolic syndrome, myocardial infarction, and all-cause mortality in individuals with diabetes (16–19). However, its relationship with stroke remains inadequately explored. Therefore, this study aims to investigate the association between UHR and stroke using data from the National Health and Nutrition Examination Survey (NHANES) 1999–2023, providing a foundation for future prospective research.

## Methods

### Population

**Figure 1:**
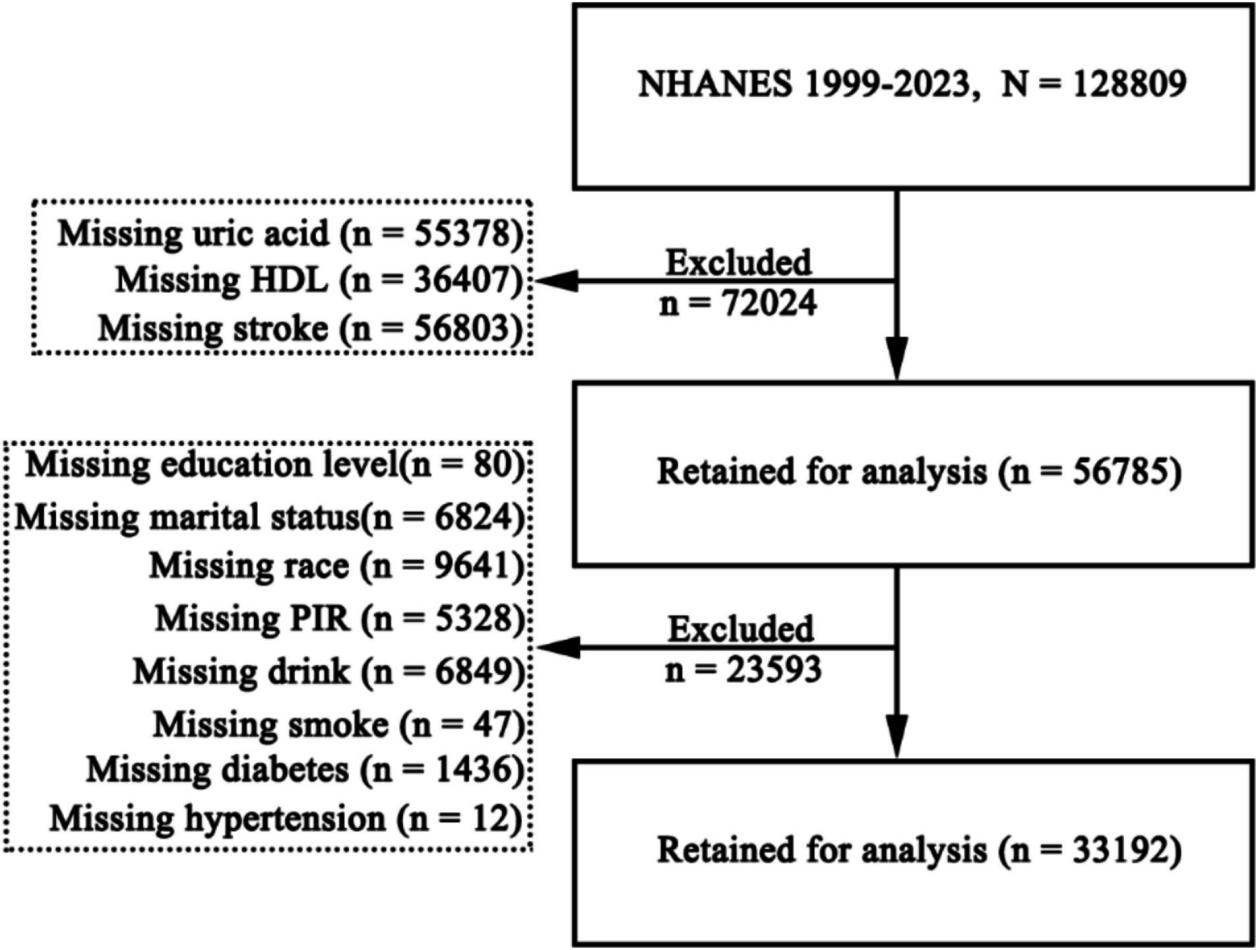
Flowchart of participant inclusion and exclusion process. NHANES, National Health and Nutrition Examination Survey; HDL, high density lipoprotein; PIR, poverty-to-income ratio.

This study included 128,809 individuals from NHANES 1999–2023. After excluding participants with missing data, 55,378 individuals lacked serum uric acid measurements, 36,407 had missing HDL data, and 56,803 had no recorded stroke status, leaving 56,785 participants eligible for further assessment. Among these, additional exclusions were made for missing data on education (*n* = 80), marital status (*n* = 6,824), ethnicity (*n* = 9,641), poverty-income ratio (PIR) (*n* = 5,328), alcohol consumption (*n* = 6,849), smoking status (*n* = 47), diabetes status (*n* = 1,436), and hypertension status (*n* = 12). After these exclusions (*n* = 23,593), a total of 33,192 individuals remained for the final analysis (Figure 1). Among the 33,192 participants, the median age was 50 years; 49.81% were female, 55.93% were White, and 1,363 (4.11%) had a history of stroke (Table 1).

**Table 1:** Clinical and demographic characteristics by stroke status.

Variables	Total (*n* = 33,192)	No stroke (*n* = 31,829)	Stroke (*n* = 1,363)	*p*
Age (year)	50.00 (35.00, 65.00)	49.00 (34.00, 64.00)	70.00 (59.00, 79.00)	<0.01
PIR	2.40 (1.22, 4.42)	2.44 (1.23, 4.48)	1.67 (1.04, 2.97)	<0.01
UR (mg/dl)	5.40 (4.50, 6.40)	5.40 (4.50, 6.40)	5.70 (4.75, 6.90)	<0.01
HDL (mg/dl)	51.00 (42.00, 62.00)	51.00 (42.00, 62.00)	48.00 (40.00, 60.00)	<0.01
UHR	10.60 (7.68, 14.38)	10.56 (7.65, 14.31)	11.75 (8.61, 16.02)	<0.01
Gender				0.96
Female	16,534 (49.81)	15,856 (49.82)	678 (49.74)	
Male	16,658 (50.19)	15,973 (50.18)	685 (50.26)	
Race				<0.01
White	18,563 (55.93)	17,735 (55.72)	828 (60.75)	
Black	8,097 (24.39)	7,728 (24.28)	369 (27.07)	
Other	6,532 (19.68)	6,366 (20.00)	166 (12.18)	
Marital				<0.01
Married	16,749 (50.46)	16,096 (50.57)	653 (47.91)	
Never married	6,640 (20.00)	6,531 (20.52)	109 (8.00)	
Separated	9,803 (29.53)	9,202 (28.91)	601 (44.09)	
Education				<0.01
<High school	6,468 (19.49)	6,018 (18.91)	450 (33.02)	
High school	7,987 (24.06)	7,620 (23.94)	367 (26.93)	
>High school	18,737 (56.45)	18,191 (57.15)	546 (40.06)	
Smoke				<0.01
Never	17,397 (52.41)	16,859 (52.97)	538 (39.47)	
Former	8,435 (25.41)	7,925 (24.90)	510 (37.42)	
Current	7,360 (22.17)	7,045 (22.13)	315 (23.11)	
Drink				<0.01
Never	4,467 (13.46)	4,246 (13.34)	221 (16.21)	
Former	5,662 (17.06)	5,178 (16.27)	484 (35.51)	
Current	23,063 (69.48)	22,405 (70.39)	658 (48.28)	
Diabetes				<0.01
No	27,678 (83.39)	26,821 (84.27)	857 (62.88)	
Yes	5,514 (16.61)	5,008 (15.73)	506 (37.12)	
Hypertension				<0.01
No	18,605 (56.05)	18,355 (57.67)	250 (18.34)	
Yes	14,587 (43.95)	13,474 (42.33)	1,113 (81.66)	

Data are presented as median (Q1, Q3) or *n* (%). Q1, 1st Quartile; Q3, 3st Quartile; PIR, Ratio of family income to poverty; UA, serum uric acid; HDL, high-density lipoprotein; UHR, the uric acid-to-HDL ratio.

### Outcome

Stroke status was determined through self-reported responses to a standardized questionnaire administered by trained researchers. Participants were asked whether they had ever been diagnosed with stroke by a physician. Those who responded “yes” were classified as having a history of stroke, while those who responded “no” were classified as not having a stroke. Participants who provided ambiguous or non-definitive responses were excluded from the analysis (20, 21).

### Calculation of UHR

In this study, the Uric Acid-to-HDL Cholesterol Ratio (UHR) was calculated using the following formula (22, 23):


$$\mathrm{UHR}=100^{*}\mathrm{UA}(\mathrm{mg}/\mathrm{dL})/\mathrm{HDL}(\mathrm{mg}/\mathrm{dL})$$

### Covariate

The covariates in this study encompassed demographic, socioeconomic, and clinical factors, including age, gender, race, education level, marital status, poverty-income ratio (PIR), smoking status, alcohol consumption, diabetes mellitus, and hypertension. Specific definitions were applied to key variables:

Diabetes mellitus was defined as meeting any of the following criteria: (1) physician-diagnosed diabetes; (2) current use of glucose-lowering medications or insulin therapy; (3) random blood glucose level >11.1 mmol/L; (4) two-hour blood glucose level >11.1 mmol/L during an oral glucose tolerance test; or (5) hemoglobin A1c level >6.5% (24, 25). Hypertension was defined as: (1) physician-diagnosed hypertension; (2) current use of antihypertensive medications; or (3) average blood pressure ≥140/90 mmHg (26). Smoking status was categorized into three groups: “Never” (fewer than 100 cigarettes smoked in a lifetime), “Former” (smoked more than 100 cigarettes but had quit), and “Current” (smoked more than 100 cigarettes and were actively smoking) (27). Alcohol consumption was classified as: “Never” (fewer than 12 instances of alcohol consumption in a lifetime), “Former” (more than 12 instances of lifetime alcohol consumption but none within the past year), and “Current” (more than 12 instances of lifetime alcohol consumption, including within the past year) (28, 29).

### Statistic

Data were extracted and analyzed using R version 4.4.1. Continuous variables were compared using either the *t*-test or the Mann–Whitney *U* test, depending on the results of normality testing. Categorical variables were analyzed using the chi-square test or Fisher’s exact test, as appropriate. The nonlinear association between UHR and stroke was evaluated using restricted cubic spline (RCS) analysis. The association between UHR and stroke risk was assessed using logistic regression models. Covariate selection was based on variance inflation factor (VIF) analysis, with variables exhibiting VIF > 10 excluded to minimize multicollinearity.

Four sequential regression models were constructed: Model 1, Unadjusted; Model 2, Adjusted for age, sex, and race; Model 3, Further adjusted for education, PIR, and marital status; Model 4: Additionally adjusted for smoking status, alcohol consumption, hypertension, and diabetes. Results were reported as odds ratio (OR) with corresponding 95% confidence interval (CI) and *p*-values. Receiver operating characteristic (ROC) curves were generated, and the area under the curve (AUC) was calculated to compare the predictive performance of the UHR with that of models using UA or HDL alone. The RCS analysis incorporated the same covariates as Model 4. Subgroup analyses were conducted using the covariates from Model 4, excluding the stratification variable. Statistical significance was defined as a two-tailed *p*-value <0.05.

### Sensitivity analysis

To assess the robustness of the findings, data were analyzed in a stratified manner. RCS analyses were conducted separately for the 1999–2004 and 1999–2010 datasets to determine whether the results were consistent with those obtained from the full 1999–2023 dataset.

## Results

### Baseline information

Table 1 presents the baseline characteristics of the study population, which included 33,192 individuals, of whom 1,363 had a prior diagnosis of stroke. The median age of the cohort was 50 years, and 49.81% were female. Participants were stratified into two groups based on stroke history, and significant differences were observed in baseline characteristics between the stroke and non-stroke groups, except for gender (*p* < 0.01). Specifically, individuals in the stroke group had a higher median age, PIR, UA, and UHR, but lower HDL levels compared to those in the non-stroke group. Additionally, the stroke group had a higher proportion of White and Black individuals, a greater proportion of separated participants, and a lower level of education. The stroke group also exhibited a lower prevalence of never-smokers, a higher proportion of former smokers and former alcohol consumers, a lower prevalence of current alcohol consumption, and a higher prevalence of hypertension and diabetes mellitus (Table 1).

Each UHR quartile (Q1–Q4) included approximately 8,300 participants. The median ages were 48, 50, 50, and 51 years, respectively. The proportions of male participants were 18.27, 41.84, 62.29, and 78.41%, and the prevalence of diabetes increased progressively from 9.26% in Q1 to 24.14% in Q4. Similarly, the prevalence of hypertension rose from 34.12 to 53.40%, and the proportion of participants with a history of stroke increased from 3.02 to 5.35%. With respect to biomarkers, the median uric acid levels across Q1–Q4 were 4.10, 5.00, 5.80, and 6.90 mg/dL, while the median HDL levels were 69.00, 55.00, 47.00, and 38.00 mg/dL. LDL levels were relatively stable across quartiles, with median values of 107.00, 112.00, 115.00, and 114.00 mg/dL (Table 2).

**Table 2:** Distribution of clinical characteristics across UHR Quartiles.

Variables	Q1	Q2	Q3	Q4	*P*
Age (year)	48.00 (34.00, 63.00)	50.00 (34.00, 65.00)	50.00 (35.00, 66.00)	51.00 (35.00, 66.00)	<0.01
PIR	2.64 (1.32, 4.82)	2.31 (1.20, 4.33)	2.39 (1.20, 4.40)	2.23 (1.18, 4.16)	<0.01
UR (mg/dl)	4.10 (3.50, 4.70)	5.00 (4.50, 5.70)	5.80 (5.20, 6.50)	6.90 (6.10, 7.70)	<0.01
HDL (mg/dl)	69.00 (60.00, 79.00)	55.00 (49.00, 62.00)	47.00 (42.00, 53.00)	38.00 (33.00, 43.00)	<0.01
UHR	6.13 (5.17, 6.94)	9.11 (8.39, 9.83)	12.24 (11.36, 13.20)	17.50 (15.78, 20.53)	<0.01
Gender					<0.01
Female	6,791 (81.73)	4,828 (58.16)	3,124 (37.71)	1791 (21.59)	<0.01
Male	1,518 (18.27)	3,473 (41.84)	5,161 (62.29)	6,506 (78.41)	<0.01
Race					<0.01
White	4,578 (55.10)	4,394 (52.93)	4,664 (56.29)	4,927 (59.38)	
Black	2,150 (25.88)	2,231 (26.88)	1990 (24.02)	1726 (20.80)	
Other	1,581 (19.03)	1,676 (20.19)	1,631 (19.69)	1,644 (19.81)	
Marital					<0.01
Married	3,940 (47.42)	3,954 (47.63)	4,306 (51.97)	4,549 (54.83)	
Never married	1745 (21.00)	1762 (21.23)	1,624 (19.60)	1,509 (18.19)	
Separated	2,624 (31.58)	2,585 (31.14)	2,355 (28.42)	2,239 (26.99)	
Education					<0.01
<High school	1,337 (16.09)	1,677 (20.20)	1,629 (19.66)	1825 (22.00)	
High school	1705 (20.52)	1980 (23.85)	2065 (24.92)	2,237 (26.96)	
>High school	5,267 (63.39)	4,644 (55.95)	4,591 (55.41)	4,235 (51.04)	
Smoke					<0.01
Never	4,939 (59.44)	4,511 (54.34)	4,128 (49.82)	3,819 (46.03)	
Former	1760 (21.18)	1926 (23.20)	2,250 (27.16)	2,499 (30.12)	
Current	1,610 (19.38)	1864 (22.46)	1907 (23.02)	1979 (23.85)	
Drink					<0.01
Never	1,199 (14.43)	1,192 (14.36)	1,070 (12.91)	1,006 (12.12)	
Former	1,074 (12.93)	1,303 (15.70)	1,510 (18.23)	1775 (21.39)	
Current	6,036 (72.64)	5,806 (69.94)	5,705 (68.86)	5,516 (66.48)	
Diabetes					<0.01
No	7,540 (90.74)	7,103 (85.57)	6,741 (81.36)	6,294 (75.86)	
Yes	769 (9.26)	1,198 (14.43)	1,544 (18.64)	2003 (24.14)	
Hypertension					<0.01
No	5,474 (65.88)	4,893 (58.94)	4,372 (52.77)	3,866 (46.60)	
Yes	2,835 (34.12)	3,408 (41.06)	3,913 (47.23)	4,431 (53.40)	
Stroke					<0.01
No	8,058 (96.98)	7,982 (96.16)	7,936 (95.79)	7,853 (94.65)	
Yes	251 (3.02)	319 (3.84)	349 (4.21)	444 (5.35)	

Data are presented as median (Q1, Q3) or *n* (%). Q1, 1st Quartile; Q3, 3st Quartile; PIR, Ratio of family income to poverty; UA, serum uric acid; HDL, high-density lipoprotein; UHR, the uric acid-to-HDL ratio. Q1, 0.74–7.68; Q2, 7.68–10.60; Q3, 10.60–14.38; Q4, 14.38–86.67. UHR, the uric acid-to-HDL ratio.

### UHR is positively associated with stroke risk

**Figure 2:**
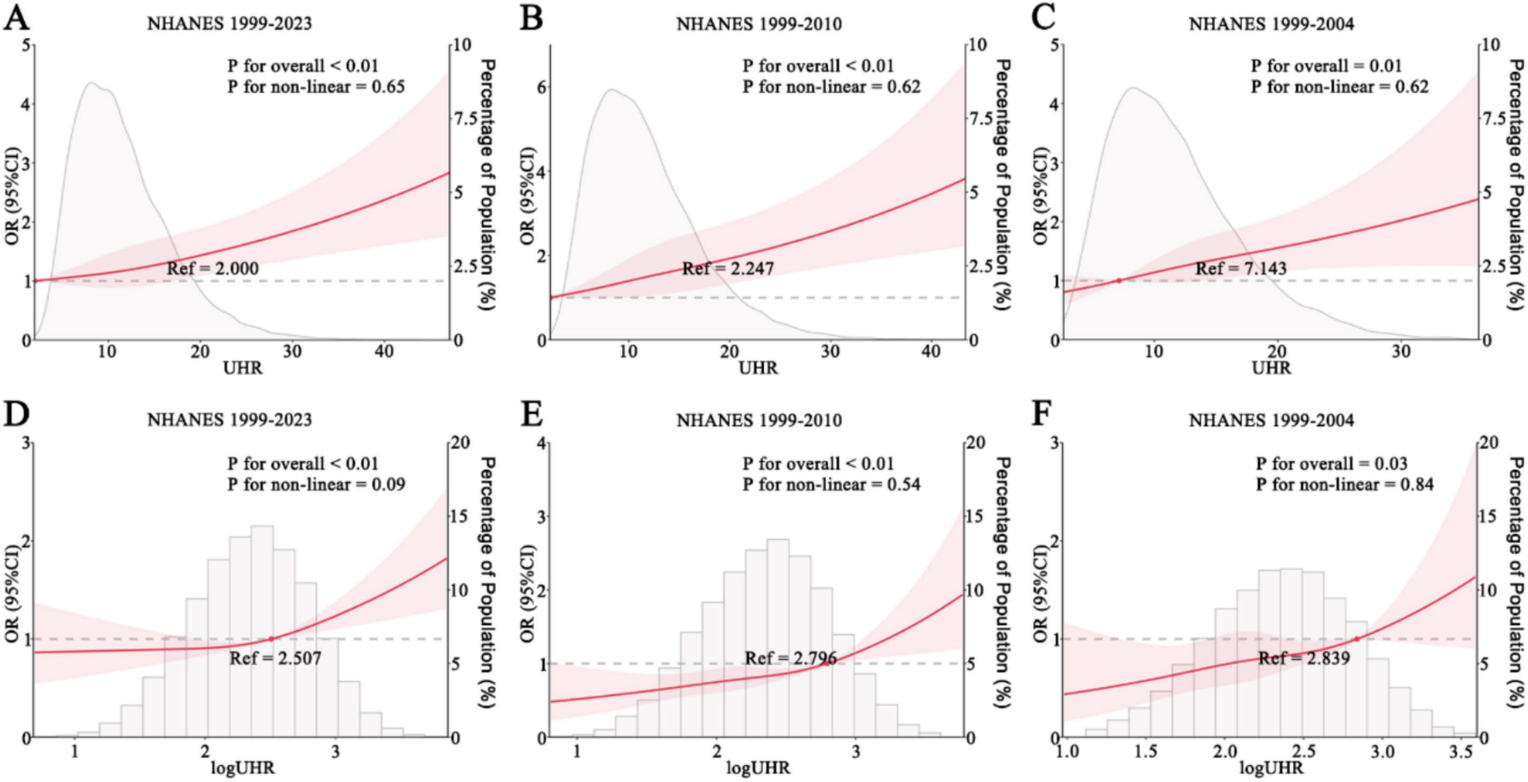
RCS analysis of UHR and stroke. **(A–C)** UHR and stroke risk in NHANES 1999–2023 **(A)**, 1999–2010 **(B)**, and 1999–2004 **(C)**. **(D–F)** log-transformed UHR and stroke risk in NHANES 1999–2023 **(D)**, 1999–2010 **(E)**, and 1999–2004 **(F)**. NHANES, National Health and Nutrition Examination Survey; UHR, uric acid-to-HDL ratio.

An initial RCS analysis of NHANES 1999–2023 data indicated no evidence of a nonlinear relationship between UHR and stroke risk (P for overall <0.01; P for nonlinearity = 0.65). The RCS curve exhibited an arc-like shape but closely approximated a straight line with a positive slope (Figure 2A). Similarly, analyses using NHANES 1999–2010 and NHANES 1999–2004 data yielded comparable results. Both datasets showed no significant nonlinear association between UHR and stroke risk (NHANES 1999–2010: *p* for overall <0.01; *p* for nonlinearity = 0.62; NHANES 1999–2004: *p* for overall = 0.01; *p* for nonlinearity = 0.62), and the corresponding RCS curves exhibited nearly identical shapes (Figures 2B,C). When the UHR was log-transformed and analyzed using RCS models, the results showed a trend similar to that observed with the untransformed UHR. However, the resulting curves displayed an approximate J-shaped pattern (Figures 2D–F).

**Figure 3:**
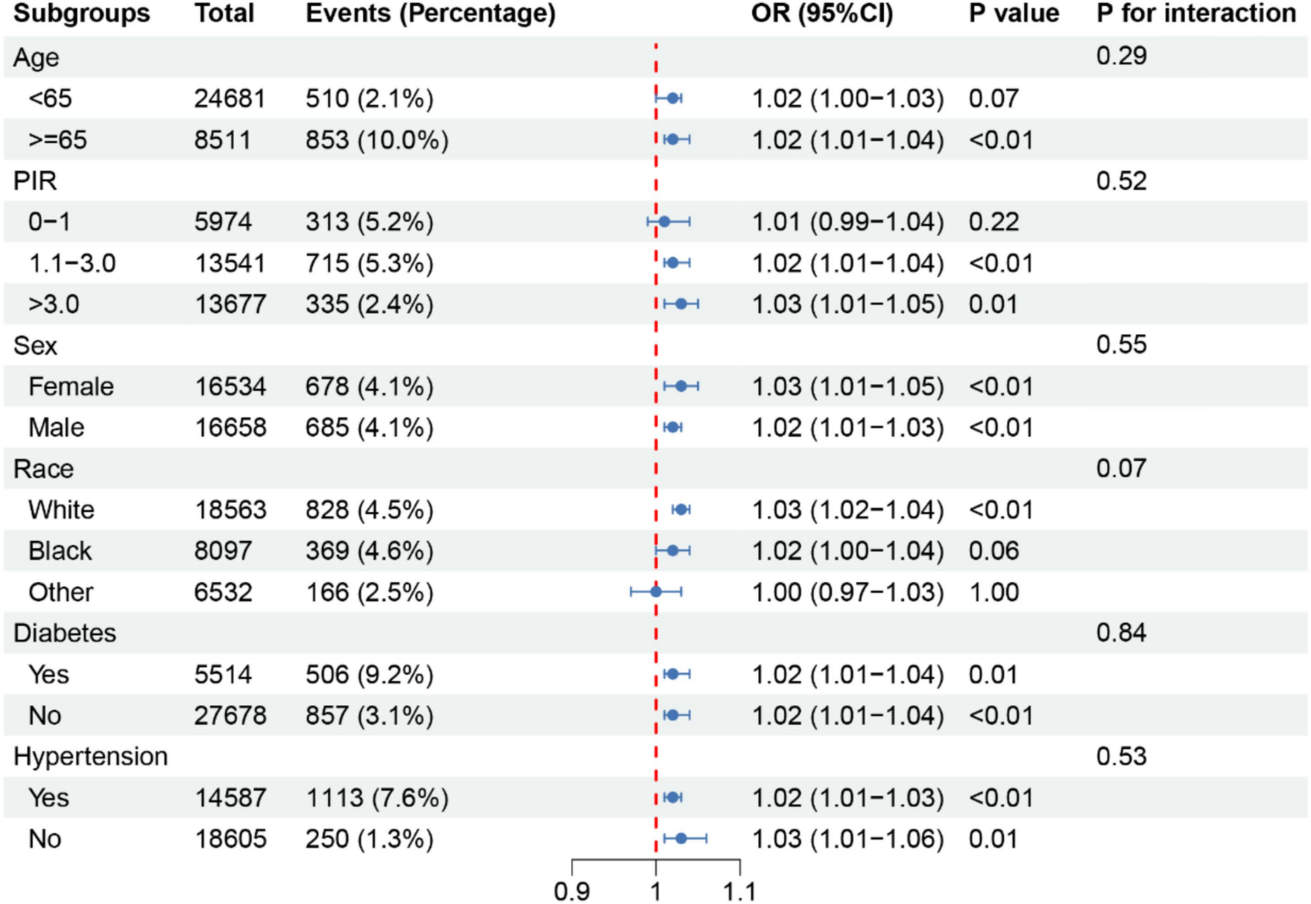
Subgroup analysis of UHR and stroke. UHR, uric acid-to-HDL ratio.

**Figure 4:**
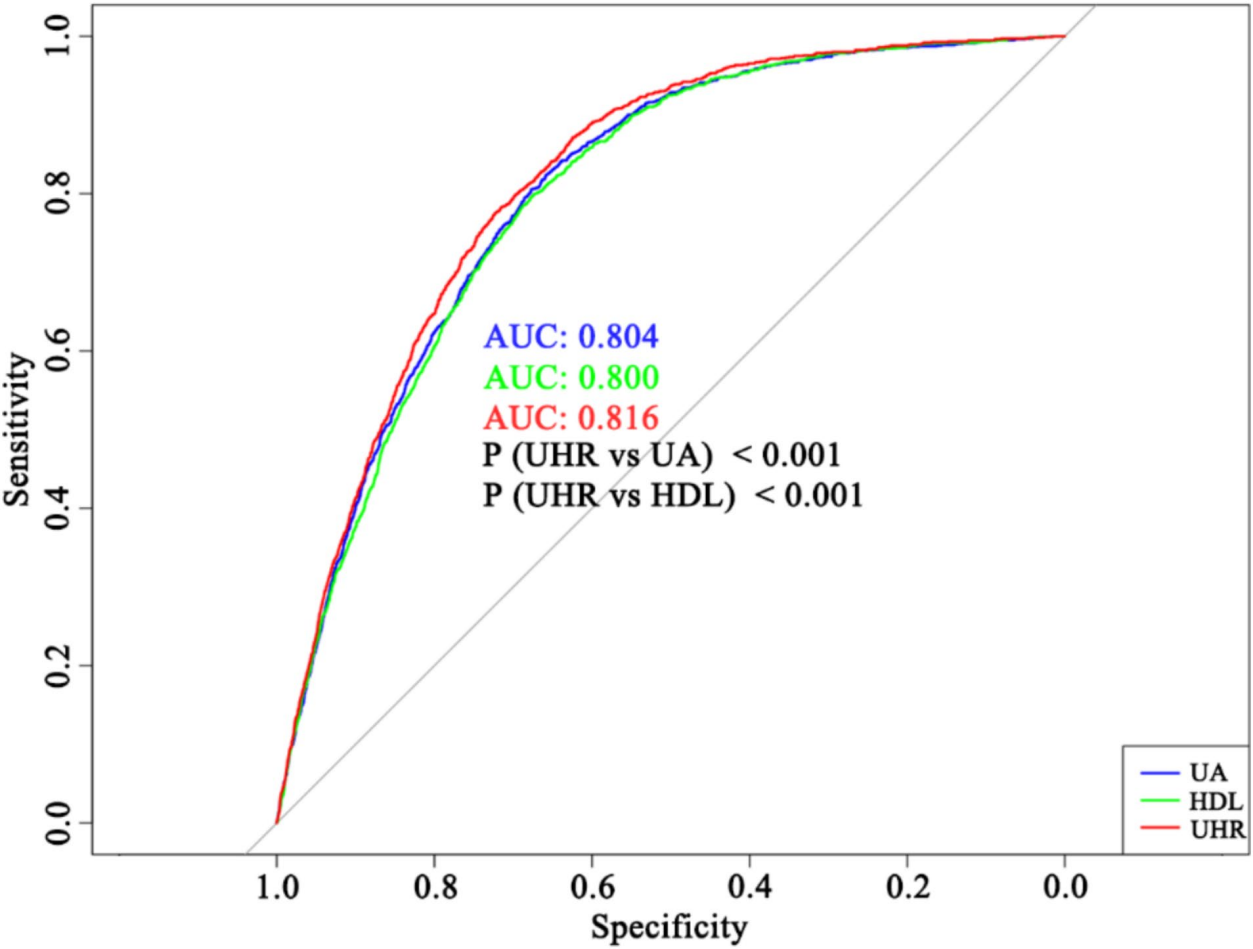
Discriminatory ability of UA, HDL, and UHR for stroke risk assessment. UA, uric acid; HDL, high density lipoprotein; UHR, uric acid-to-HDL ratio; AUC, area under curve. *p*-values were calculated using DeLong’s test for correlated ROC curves.

Subsequently, logistic regression analysis was performed to evaluate the association between UHR and stroke risk. When UHR was analyzed as a continuous variable, the results indicated a significant positive association with stroke risk across all models (Model 1: OR = 1.04, 95% CI 1.03–1.05, *p* < 0.01; Model 2: OR = 1.05, 95% CI 1.04–1.06, *p* < 0.01; Model 3: OR = 1.04, 95% CI 1.03–1.05, *p* < 0.01; Model 4: OR = 1.02, 95% CI 1.01–1.03, *p* < 0.01) (Table 2). Subgroup analyses demonstrated that in most subgroups, UHR remained positively associated with stroke risk, except for individuals classified as “Other Race.” Specifically, the association remained significant among individuals aged ≥65 years (OR = 1.02, 95% CI 1.01–1.04, *p* < 0.01), those with a PIR of 1.1–3.0 (OR = 1.02, 95% CI 1.01–1.04, *p* < 0.01) or PIR > 3.0 (OR = 1.03, 95% CI 1.01–1.05, *p* = 0.01), females (OR = 1.03, 95% CI 1.01–1.05, *p* < 0.01), males (OR = 1.02, 95% CI 1.01–1.03, *p* < 0.01), White individuals (OR = 1.03, 95% CI 1.02–1.04, *p* < 0.01), individuals with diabetes (OR = 1.02, 95% CI 1.01–1.04, *p* = 0.01) or without diabetes (OR = 1.02, 95% CI 1.01–1.04, *p* < 0.01), and individuals with hypertension (OR = 1.02, 95% CI 1.01–1.03, *p* < 0.01) or without hypertension (OR = 1.03, 95% CI 1.01–1.06, *p* = 0.01) (Figure 3). However, the association was not statistically significant among individuals aged <65 years (OR = 1.02, 95% CI 1.00–1.03, *p* = 0.07), those with a PIR of 0–1.0 (OR = 1.01, 95% CI 0.99–1.04, *p* = 0.22), Black individuals (OR = 1.02, 95% CI 1.00–1.04, *p* = 0.06), and those classified as “Other Race” (OR = 1.00, 95% CI 0.97–1.03, *p* = 1.00) (Figure 3). ROC curve analysis demonstrated that the model incorporating UHR yielded an AUC of 0.816, which was slightly higher than that of models using UA (AUC = 0.804) or HDL (AUC = 0.800) alone (Figure 4). DeLong’s test showed that UHR had significantly higher AUC than UA (*p* < 0.001) and HDL (*p* < 0.001) for predicting stroke risk (Figure 4).

When UHR was categorized into quartiles (Q1–Q4) with Q1 as the reference, Q2–Q4 were significantly associated with an increased risk of stroke across Models 1–3. However, in the fully adjusted Model 4, only Q4 remained significantly associated with a higher stroke risk (OR = 1.31, 95% CI 1.11–1.55, *p* < 0.01), whereas Q2 (OR = 1.10, 95% CI 0.93–1.30, *p* = 0.26) and Q3 (OR = 1.11, 95% CI 0.94–1.31, *p* = 0.23) were not statistically significant (Table 3). When Q1-Q3 were used as the reference group, Q4 was significantly associated with an increased risk of stroke (Model 4: OR, 1.21; 95% CI, 1.07–1.37; *p* < 0.01), a finding consistent across all models (Table 4).

**Table 3:** Logistic regression analysis of UHR and stroke.

Model	Continuous	Quartiles of UHR index			
		Q1	Q2	Q3	Q4
Model 1	1.04 (1.03, 1.05) *p* < 0.01	ref	1.28 (1.08, 1.52) *p* < 0.01	1.41 (1.20, 1.67) *p* < 0.01	1.82 (1.55, 2.13) *p* < 0.01
Model 2	1.05 (1.04, 1.06) *p* < 0.01	ref	1.27 (1.08, 1.50) *p* < 0.01	1.41 (1.20, 1.67) *p* < 0.01	1.89 (1.60, 2.23) *p* < 0.01
Model 3	1.04 (1.03, 1.05) *p* < 0.01	ref	1.21 (1.03, 1.42) *p* = 0.02	1.33 (1.13, 1.57) *p* < 0.01	1.71 (1.45, 2.02) *p* < 0.01
Model 4	1.02 (1.01, 1.03) *p* < 0.01	ref	1.10 (0.93, 1.30) *p* = 0.26	1.11 (0.94, 1.31) *p* = 0.23	1.31 (1.11, 1.55) *p* < 0.01

Q1, 0.74–7.68; Q2, 7.68–10.60; Q3, 10.60–14.38; Q4, 14.38–86.67. UHR, the uric acid-to-HDL ratio.

**Table 4:** Logistic regression analysis of UHR and stroke.

Model	Q1–Q3	Q4	
		OR (95% CI)	*p*
Model 1	ref	1.48 (1.31, 1.66)	<0.01
Model 2	ref	1.51 (1.34, 1.70)	<0.01
Model 3	ref	1.42 (1.26, 1.60)	<0.01
Model 4	ref	1.21 (1.07, 1.37)	<0.01

Q1, 0.74–7.68; Q2, 7.68–10.60; Q3, 10.60–14.38; Q4, 14.38–86.67. UHR, the uric acid-to-HDL ratio.

## Discussion

By analyzing data from NHANES 1999–2023, the present study confirms a positive association between UHR and stroke risk, with this relationship remaining significant across most subgroups. Furthermore, stratified analyses provided additional validation, demonstrating consistent and robust results.

UHR was initially introduced as a marker for predicting metabolic syndrome and glycemic control in individuals with type 2 diabetes mellitus, with higher UHR levels indicating an increased risk of metabolic syndrome or poor diabetes control. Additionally, UHR has been strongly associated with visceral fat accumulation in patients with diabetes (16, 30, 31). In subsequent research, UHR has been linked to the development of cardiovascular disease. A Korean cohort study demonstrated a positive association between elevated UHR and the incidence of ischemic heart disease in individuals without diabetes (32). Notably, the findings of that study align with aspects of the present study, as multivariable-adjusted regression analyses indicated that only the highest UHR quartile (Q4) exhibited an increased risk relative to Q1 (Q4 hazard ratio [HR] = 1.57, 95% CI 1.01–2.45). However, Kaplan–Meier survival analysis in that study suggested that higher UHR levels were associated with a greater risk of ischemic heart disease (32). Beyond its role in ischemic heart disease, UHR has been independently associated with plaque rupture, erosion, and thrombosis in patients with acute coronary syndrome, demonstrating superior predictive value compared to low density lipoprotein (13). Furthermore, UHR has been linked to the severity of ischemic heart disease; elevated UHR has been associated with the diagnosis of stable and unstable angina, as well as poor collateral circulation in patients with chronic total coronary occlusion (33). Additionally, increased UHR has been negatively correlated with fractional flow reserve in individuals with moderate coronary artery stenosis (34). In a study of U. S. adults, UHR was strongly associated with both all-cause and cardiovascular mortality, with adjusted hazard ratios in the highest quintile of 1.16 (95% CI 1.05–1.29) for all-cause mortality and 1.20 (95% CI 1.00–1.45) for cardiovascular mortality (35).

Although no studies have directly examined the association between UHR and stroke, several investigations have explored the relationship between UHR and cardiovascular diseases, including stroke. A study conducted in Shanghai reported a positive association between UHR and overall cardiovascular disease risk (OR = 1.28, 95% CI 1.02–1.61) (36). Additionally, a study of 566 patients with chronic total occlusion followed for a median of 43 months found that elevated UHR was significantly associated with major adverse cardiovascular events, with a HR of 2.01 (95% CI 1.62–2.49) per standard deviation increase in UHR (37). Regarding stroke risk factors, in addition to diabetes mellitus, as previously discussed, UHR has been identified as an independent risk factor for poor blood pressure control in individuals with hypertension. Specifically, each one-unit increase in UHR was associated with a 7.3-fold higher risk of uncontrolled hypertension (*p* < 0.001, 95% CI 3.90–13.63) (38). Furthermore, UHR has been independently linked to an increased risk of atrial fibrillation (OR = 1.010, 95% CI 1.007–1.013, *p* < 0.001), demonstrating superior predictive value compared to UA and HDL alone (39). In the present study, individuals in the stroke group exhibited higher UHR levels, along with a greater prevalence of diabetes mellitus and hypertension, findings that align with the results of these previous studies.

UHR is derived from UA and HDL, making it inherently positively correlated with UA and negatively correlated with HDL. A Mendelian randomization study demonstrated that UA is causally associated with large artery atherosclerotic ischemic stroke and small artery occlusive ischemic stroke, but not with cardioembolic stroke (40). Additionally, UA has been linked to an increased risk of spontaneous hemorrhagic transformation in male patients with ischemic stroke (OR = 1.85, 95% CI 1.07–3.19, *p* = 0.028) (41). A meta-analysis further highlighted that elevated UA levels were significantly associated with stroke recurrence, with a pooled OR of 1.80 (95% CI 1.47–2.20, *p* < 0.001) (42). In contrast, the relationship between HDL and stroke appears more complex, with studies suggesting a *U*-shaped association between HDL levels and stroke risk (P for nonlinearity < 0.001). Specifically, stroke risk was lowest at an HDL-C level of 1.29 mmol/L, with both lower and higher cumulative HDL-C levels associated with an increased risk of ischemic and hemorrhagic stroke (43). This *U*-shaped relationship limits the predictive utility of HDL alone in stroke risk assessment. Conversely, the present study found a nearly linear relationship between UHR and stroke, suggesting that UHR may serve as a more practical and intuitive marker, as higher UHR values consistently corresponded to a greater stroke risk. If validated by prospective studies, UHR may offer superior predictive value for stroke compared to UA or HDL alone, given its straightforward interpretation and ease of calculation. In conclusion, this study underscores the positive association between UHR and stroke risk. However, further prospective research is needed to investigate the role of UHR in stroke onset, recurrence, and stroke-related mortality.

This study has several limitations. First, as a cross-sectional study, it can only establish associations rather than causal relationships between UHR and stroke risk. Second, UHR was assessed at a single time point, without accounting for longitudinal changes or cumulative effects over time. Third, stroke diagnosis in this study was based on self-reported data, which may introduce recall bias or misclassification. Additionally, information on stroke subtypes and treatment strategies was not available, limiting further stratified analyses. Finally, since the study population was primarily derived from the U. S., caution is warranted when generalizing these findings to other populations.

## Conclusion

In this study population, an elevated UHR was positively associated with an increased risk of stroke. However, further research, particularly prospective studies, is needed to better elucidate the relationship between UHR and stroke risk, including its potential role in stroke prediction and prevention.

## Data Availability

Publicly available datasets were analyzed in this study. This data can be found here: https://www.cdc.gov/nchs/nhanes/.
